# High prevalence of soil-transmitted helminths and schistosomiasis among primary schoolchildren in Southwest Ethiopia: the need for health strategies alongside mass drug administration

**DOI:** 10.1093/inthealth/ihad083

**Published:** 2023-11-03

**Authors:** Asrat Meleko, Dorin Brener Turgeman, Naomi Caplan, Sarit Baum, Nisan K Zerai, Willemijn Zaadnoordijk, Michal Bruck, Galia Sabar, Zvi Bentwich, Rachel Golan

**Affiliations:** NALA, Carlebach 29, Tel Aviv-Yafo 6713224, Israel; Department of Public Health, Mizan Tepi University College of Medicine and Health Sciences, Tepi, 5160, Ethiopia; NALA, Carlebach 29, Tel Aviv-Yafo 6713224, Israel; NALA, Carlebach 29, Tel Aviv-Yafo 6713224, Israel; NALA, Carlebach 29, Tel Aviv-Yafo 6713224, Israel; NALA, Carlebach 29, Tel Aviv-Yafo 6713224, Israel; Merck, Frankfurter Strasse 250, Darmstadt 64293 Germany; NALA, Carlebach 29, Tel Aviv-Yafo 6713224, Israel; The Department of Middle Eastern and African History, Tel Aviv University, Tel Aviv-Yafo P.O. Box 39040, Israel; NALA, Carlebach 29, Tel Aviv-Yafo 6713224, Israel; Shraga Segal Department of Microbiology, Immunology and Genetics, Ben-Gurion University of the Negev, Beer Sheva P.O. Box 653, Israel; NALA, Carlebach 29, Tel Aviv-Yafo 6713224, Israel; Department of Epidemiology, Biostatistics and Community Health Sciences, Ben-Gurion University of the Negev, Beer Sheva P.O. Box 653, Israel

**Keywords:** behaviors, health education, mass drug administration, schistosomiasis, soil-transmitted helminths

## Abstract

**Background:**

Soil-transmitted helminths (STH) and schistosomiasis remain widely prevalent in Ethiopia. The aim of this study was to evaluate the prevalence of STH and schistosomiasis among schoolchildren in Gidi Bench district (Southern Nations, Nationalities, and People's Republic, Southwest Ethiopia) and the association with knowledge and health-related behaviors.

**Methods:**

A cross-sectional study was conducted. Stool samples, analyzed by the Kato-Katz technique and a knowledge, attitudes and practices questionnaire, were collected.

**Results:**

Out of 611 participants (mean age 12.8±3.1 y), 129 (21.1%) were infected with schistosomiasis and 382 (62.5%) had STH. More than 30% (n=195, 31.9%) were infected with a single intestinal parasite, while 138 (22.6%) and 47 (7.7%) were infected with two or three parasitic infections, respectively. Boys and those who did not participate in school clubs had higher infection rates (p=0.05). Lower parasitic infection was associated with using a latrine when available, washing hands and vegetables and wearing shoes regularly. Higher rates of infection were found among those who reported swimming and washing cloths and utensils in the river regularly.

**Conclusions:**

Schistosomiasis and STH were highly prevalent among schoolchildren in Gidi Bench district. Infection rates were associated with gender, lack of knowledge on parasitic infections and unhealthy behaviors. Findings from this study may assist in decision making regarding disease prevalence and methods of control alongside mass drug administration.

## Introduction

Infections caused by soil-transmitted helminths (STH) are among the most common infections in low-income countries, with >1.5 billion people infected worldwide with STH, mostly in tropical and subtropical regions.^1^ The most common STH infections in sub-Saharan Africa are attributable to *Ascariasis lumbricoides*, hookworms and *Trichiuris trichiura*.^2^ Schistosomiasis, blood flukes of the genus *Schistosoma*, is a parasitic disease that affects an estimated 240 million people worldwide.^3^ It is the second most common parasitic disease, killing an estimated 280 000 people each year in the African region alone, where >90% of the cases occur.^4^ The high prevalence of intestinal parasitic transmission and schistosomiasis in developing countries is attributed to poverty-related factors including poor sanitation, a lack of proper hygiene, a scarcity of clean water, open-field defecation, a lack of adequate health services and a low level of awareness.^5,6^

In Ethiopia, approximately 5 million people are infected with intestinal schistosomiasis, with an additional 37.5 million at risk.^2^  *Schistosoma mansoni* and *Schistosoma heamatobium* are the two species found in Ethiopia, with *S. mansoni* being the most prevalent. STH infections are the second most common cause of outpatient morbidity in the country. Drinking water and latrine coverage are limited in Ethiopia and therefore intestinal parasites and other communicable diseases are widespread in many communities.^7,8^

Since 2015, mass drug administration (MDA) for STHs and schistosomiasis among school-age children has been implemented through school-based deworming programs in most regions of Ethiopia.

While MDA efforts for the control of neglected tropical diseases (NTDs) have made a substantial impact, preventing reinfection in communities remains difficult without also improving water, sanitation and hygiene (WASH) conditions and practices.^9^ Despite some improvements in the WASH status in Ethiopia, long-term prevention and control of NTDs has not been achieved in all communities.^10,11^ The aim of this study was to assess the prevalence of schistosomiasis and STH and examine the association between these diseases and knowledge, attitudes and practices (KAP) of schoolchildren in the newly established Gidi Bench district, of Bench Sheko Zone, SouthWest Ethiopia Peoples’ Region (SWEPR).

## Methods and Subjects

### Study design, setting and population

A school-based cross-sectional study was conducted from May 2021 to August 2021 in the newly established Gidi Bench district of Bench Sheko Zone, Southern Nations, Nationalities, and People's Republic . The district is divided into seven kebeles (i.e. the smallest administrative unit, equivalent to a village or a neighborhood), with Dizu serving as the district capital. According to the most recent census, the current population of the district is approximately 45 000 people.^12^ The study population for this study included all students from preprimary school (kindergarten) through grade 8 who attended public primary schools in Gidi Bench district.

### Data collection procedure and tools

A single population proportion formula was used to calculate the minimum sample size of study participants, assuming a prevalence of 50% (p), considering 5% margin of error (d) and 95% CI level. This was then multiplied by a design effect of 1.5 with an added 10% contingency for the non-response rate. The schools were classified into clusters based on their kebele. Schools were chosen at random from each strata. Using the student's attendance list as a sampling frame, students were randomly selected from each school.

A structured questionnaire in Amharic was used to collect data on KAP of the students regarding the relevant diseases assessed. Questionnaires were self-administered under the supervision of local extension workers, to ensure data quality. The consistency and completeness of each questionnaire was evaluated both in the field and before data entry. Each student who answered a KAP questionnaire provided a stool sample.

### Stool sample collection and parasitological examination

A single stool specimen was collected from each study participant using a clean, dry and leak-proof stool cup labeled with an identification number. Students were instructed by the school director on how to provide a thumb-sized stool specimen. The samples were collected at each school and transported to Mizan-Tepi University's Clinical Laboratory. The Kato Katz technique was used for the assessment of stool samples. The Kato Katz samples were prepared using a 41.7-g template. Each smear was examined within 30–60 min of preparation to avoid the hookworm disintegrating. The prevalence of major STHs such as roundworms (*Ascaris lumbricoides*), whipworms (*Trichuris trichiura*) and hookworms was assessed.

### Statistical analysis

Participants' characteristics were analyzed using descriptive analysis. ANOVA and the t test were used to examine the associations between sociodemographic characteristics and the infection rate. Multivariable logistic regressions were used to identify factors associated with infection. p<0.05 was considered significant. The EpiData version 3.1 (EpiData Association, Odense, Denmark) statistical package was used to enter the data; IBM SPSS Statistics for Windows Version 25.0 (IBM Corp. IBM SPSS Statistics for Windows, Armonk, NY, USA) was used for analysis.

## Results

A total of 611 enrolled schoolchildren were included in the study (boys, n=330 [54.0%]; girls, n=281 [46%]). The age of study participants ranged from 7 to 18 y, with a mean of 12.8 (3.1) y. Two hundred and eighty-three (46%) schoolchildren attended grades 1–4 and 323 (54%) schoolchildren attended grades 5–8. The study sample is not representative by age. Intestinal parasites were present in the samples of 382 (62.5%) students, with boys presenting higher rates of infection compared with girls (boys with positive sample: n=234 [70.9]; girls with positive sample: n=148 [52.7%]; p>0.001). *Trichuris trichuria* and *A. lumbricoides* were the most common intestinal parasites found, accounting for 255 (41.7%) and 204 (33.4%) of all positive cases, respectively. *Schistosoma mansoni* infection was found in 129 (21.1%) students (Table 1). Other species were not detected in the study sample. The majority of positive cases (n=195 [31.9%]) were infected with a single intestinal parasite, while 138 (22.6%) and 47 (7.7%) were infected with two and three parasites, respectively. Schoolchildren who did not participate in school WASH or health clubs had higher infection rates (positive sample among children attending clubs: n=117 vs positive samples among children not attending school clubs; p=0.05).

**Table 1.** Prevalence of infection by sex of schoolchildren in the Gidi Bench district, Bench Sheko zone, SWEPR, Ethiopia (n=611)

	Boys, no.	%	Girls, no.	%	Total	%
Parasite species						
*S. mansoni*	81	24.5	48	17.1	129	21.1
*A. lumbricoides*	117	35.5	87	31.0	204	33.4
*T. trichura*	168	50.9	87	31.0	255	41.7
*Hookworm*	7	2.1	7	2.5	14	2.3
Infection presence						
No infection	101	29.5	140	48.1	241	38.1
Single infection	116	33.9	84	28.9	200	38.1
Double infection	95	27.8	47	16.2	142	22.4
Multiple infection	30	8.8	20	6.9	50	7.9

All students completed a KAP questionnaire, which included three sections, consisting of knowledge (regarding diseases), attitudes (towards diseases) and practices (of health behaviors).

Knowledge: About one-half of the participants, 309 (50.6%), had never heard of intestinal parasites. More than one-half, 332 (54.3%), agreed that walking barefoot does not transmit intestinal parasites and 336 (55.0%) thought diarrhea was not a symptom of intestinal parasites. Only 102 (16.7%) of the students knew that schistosomiasis was transmitted through contact with contaminated water (Supplementary Table S1).

Attitudes: A total of 219 (35.8%) participants did not think it was necessary to wear shoes and 237 (38.8%) participants thought it was acceptable for children to defecate outside. More than one-half of the students (328 [53.7%]) believed that not washing their hands causes illness (Supplementary Table S2).

Practices: More than 40% of students (n=249, 40.8%) reported swimming, playing and washing in the rivers regularly, with the majority being boys (boys: n=288, girls: n=219; p=0.005) (Supplementary Table S3). In addition, 346 students (57%) reported they did not wash their hands before eating and 354 (57.9%) reported they did not wash their hands after using the latrines.

The association between KAP and infection was examined (Table 2). Lower parasitic infection was associated with using a latrine when available, washing hands and vegetables and wearing shoes regularly. Higher rates of infection were found among those who reported swimming and washing cloths and utensils in the river regularly. No association was found between those who reported washing hands before eating and infection rates (Table 2, Supplementary Table S4).

**Table 2.** Factors associated with parasitic infection in schoolchildren in the Gidi Bench district, Bench Sheko zone, SWEPR, Ethiopia (n=611)

Variable	B	SE	Exp (B)	p
Constant	2.29	0.69	9.76	0.001
Sex (boys)	0.85	0.18	2.34	<0.001
Participation in school clubs (WASH or health)	−0.52	0.19	0.59	0.008
Wear shoes regularly	0.30	0.13	1.36	0.024
Wash hands when dirty	0.57	0.19	1.77	0.003
Use latrine when available	−0.68	0.14	0.53	<0.001
Treat vegetables before consumption	−0.37	0.14	0.69	0.007
Use river regularly (swim/play/wash)	0.35	0.12	1.42	0.005
Wash cloths and utensils in river	−1.05	0.18	0.35	<0.001

Abbreviation: WASH, water, sanitation and hygiene.

## Discussion

Findings from this study indicate that both schistosomiasis and STH infections are widespread and a major public health concern in the newly established Gidi Bench district of Bench Sheko Zone. Our findings demonstrate the importance of knowledge and behavior in contributing to the high prevalence of these infections.

Our study has some limitations. First, seasonality of parasitic infections was not evaluated and therefore prevalence of disease as measured in this study does not consider this factor. Second, we did not use precision mapping for sampling the study population. This might have led to bias in our study sample.^13^ The KAP questionnaire was self-administrated by relatively young children (aged <10 y). Although this was under the supervision of trained local extension workers, the data collected might have been biased. Also, we did not conduct focus group discussions. However, several studies have shown an association between KAP questionaries and parasitic infection in other countries in the region.^14,15^ The strengths of the study include the relatively large sample and the ability to collect both specimens and questionaries from young children in rural communities.

Less than one-half of the students thought walking barefoot is an unhealthy behavior or that diarrhea can be a symptom of a disease. Only a few knew infection could occur while swimming in the rivers. Moreover, a lack of knowledge regarding vegetable handling, swimming in rivers, using latrines, washing clothing and utensils in open water sources and washing hands after defecation were all associated with parasitic infection. These findings are consistent with other studies conducted in Ethiopia where a lack of knowledge regarding parasitic infections as well as practices of washing in open water sources, open field defecation and eating untreated raw vegetables were all associated with high prevalence of intestinal parasites.^16–18^ Knowledge regarding endemic diseases and ways to stop transmission are critical for eliminating these infections,^19^ yet less than one-half of the students in this study were aware of intestinal parasites. These gaps in knowledge could be attributed to differences in the status of health education in schools and participation in WASH or health clubs in the schools.^20^

Schistosomiasis is a water-borne disease. In this study, >40% of the students reported swimming, playing and washing in rivers regularly. Frequent use of the rivers was also found in other studies conducted in Ethiopia, ranging from 8% to 38%.^14,21,22^ Increasing the knowledge of the students regarding the route of the disease transmission and the association between the rivers and the disease may encourage children to use the water sources in a safer manner. However, because these rivers are usually the only source of water available for the community, implementing vector control with the community should also be considered.^23^

Boys had a higher prevalence of intestinal parasites compared with girls in the age range of this study. These findings are consistent with findings from rural communities in Northwest Ethiopia^13,24^ and could be attributed to the fact that boys use rivers for daily activities more often than girls do. This finding should be considered when promoting healthy behaviors. Also, students who did not participate in school clubs had higher infection rates. This could be attributed to the roles that these students hold in disease prevention and health promotion activities among their peers.

### Conclusions

Schistosomiasis and intestinal parasitic infections were highly prevalent among schoolchildren in Gidi Bench district and were associated with age, sex, lack of knowledge on parasitic infections and unhealthy behaviors. Findings from this study may assist in decision making and serve as a guide for developing more targeted and relevant interventions in these communities. These findings may be used to better understand the context within the community and the relationship between knowledge, behavior and disease prevalence.

## Supplementary Material

ihad083_Supplemental_File

## Data Availability

The data underlying this research article is not publicly available, but can be provided by the corresponding author upon request.
