# Supplementary material for: High prevalence of soil-transmitted helminths and schistosomiasis among primary schoolchildren in Southwest Ethiopia: the need for health strategies alongside mass drug administration
Source: Int Health. 2023 Nov 3;16(5):529–33. doi: 10.1093/inthealth/ihad083 (PMC11375566; doi:10.1093/inthealth/ihad083)
Supplement: ihad083_Supplemental_File [file ihad083_supplemental_file.docx]

Meleko et al.

High prevalence of soil-transmitted helminths and schistosomiasis

among primary schoolchildren in Southwest Ethiopia: the need for

health strategies alongside mass drug administration

**Supplemental Materials**

Table S1. Knowledge of students regarding preventive behaviors for Schistosomiasis and Intestinal parasites at Gidi Bench district, Bench sheko zone, SWEPR, Ethiopia, n=611

| Variable | Category | Frequency | Percentage (%) |
| --- | --- | --- | --- |
| Heard about intestinal parasites before | Yes | 302 | 49.4 |
|  | No | 309 | 50.6 |
| Walking barefoot causes intestinal infection | Yes | 279 | 45.7 |
|  | No | 332 | 54.3 |
| Diarrhea is symptom of intestinal parasitic infection | Yes | 275 | 45.0 |
|  | No | 336 | 55.0 |
| Runny nose is symptom of intestinal parasitic infection | Yes | 320 | 52.4 |
|  | No | 291 | 47.6 |
| How to handle vegetables before consumption, to prevent infections | Put them in the sun | 95 | 15.5 |
|  | Wash, cook or peel vegetables | 290 | 47.5 |
|  | Do nothing | 226 | 37.0 |
| Can open defecation cause disease | Yes | 425 | 69.6 |
|  | No | 186 | 30.4 |
| How is Schistosomiasis transmitted | I don’t know | 143 | 23.4 |
|  | Eating contaminated food | 145 | 23.7 |
|  | Dirty hands | 99 | 16.2 |
|  | Swimming or bathing in infested water | 102 | 16.7 |
|  | Playing with soil | 122 | 20.0 |

Table S2. Attitudes of students towards preventive behaviors for Schistosomiasis and intestinal parasitic in primary schools of Gidi Bench district, Bench Sheko zone, SWEPR, Ethiopia, n=611

| Variable | Category | Frequency | Percentage (%) |
| --- | --- | --- | --- |
| Wearing shoes is important for my health | Agree | 392 | 64.2 |
|  | Disagree | 219 | 35.8 |
| Intestinal parasites are a big health problem in my school | Agree | 295 | 48.3 |
|  | Disagree | 316 | 51.7 |
| It is not okay if children defecate in the water or the field | Agree | 374 | 61.2 |
|  | Disagree | 237 | 38.8 |
| It is better to use latrine | Agree | 349 | 57.1 |
|  | Disagree | 262 | 42.9 |
| I feel embarrassed if my friends see me open defecate | Agree | 399 | 65.3 |
|  | Disagree | 212 | 34.7 |
| If you fail to wash your hands you will be exposed to disease | Agree | 328 | 53.7 |
|  | Disagree | 283 | 46.3 |
| I believe that intestinal parasites can be prevented | Agree | 299 | 48.9 |
|  | Disagree | 312 | 51.1 |

Table S3. Practice of study participants towards preventive behaviors for Schistosomiasis and intestinal parasitic at Gidi Bench district, Bench Sheko zone, SWEPR, Ethiopia, n=611

| Variable | Category | Frequency | Percentage (%) |
| --- | --- | --- | --- |
| I swim, play or wash myself in the river | Never | 123 | 20.1 |
|  | Sometimes | 239 | 39.1 |
|  | Everyday | 249 | 40.8 |
| If a latrine is available, I use it | Never | 104 | 17.0 |
|  | Sometimes | 153 | 25.0 |
|  | All the time | 354 | 57.9 |
| I wear shoes | Never | 104 | 17.0 |
|  | Sometimes | 168 | 27.5 |
|  | All the time | 339 | 55.5 |
| I wash clothes or utensils in open water sources | Yes | 363 | 59.4 |
|  | No | 248 | 40.6 |
| I wash my face when it is dirty | Yes | 297 | 48.6 |
|  | No | 314 | 51.4 |
| Wash hands when they look or feel dirty | Yes | 309 | 50.6 |
|  | No | 313 | 51.2 |
| Wash hands before eating or preparing food | Yes | 265 | 43.4 |
|  | No | 346 | 56.6 |
| Wash hands after defecation | Yes | 257 | 42.1 |
|  | No | 354 | 57.9 |
| Wash hands after play | Yes | 103 | 16.9 |
|  | No | 508 | 83.1 |

Table S4. Factors associated with intestinal parasites infection in primary schoolchildren, Gidi Bench district, Bench Sheko zone, SWEPR, Ethiopia, n=611

| Variable | Category | IP Infection Status | | Total | AOR (95 % CI) |
| --- | --- | --- | --- | --- | --- |
|  |  | Positive | Negative |  |  |
|  |  | n (%) | n (%) | n (%) |  |
| Sex | Male | 234 (61.3) | 96 (41.9) | 330 (54.0) | 2.48 (1.687, 3.635) |
|  | Female | 148 (38.7) | 133 (58.1) | 281 (46.0) | 1 |
| How do you treat vegetables before consumption? | Wash, cook or peel vegetables | 165 (43.2) | 125 (54.6) | 290 (47.5) | 1 |
|  | Don't know the correct answer | 217 (56.8) | 104 (45.4) | 321 (52.5) | 1.82 (1.243, 2.667) |
| It is acceptable for young children to defecate openly | Agree | 245 (64.1) | 129 (56.3) | 374 (61.2) | 2.57 (1.615, 4.091) |
|  | Disagree | 137 (35.9) | 100 (43.7) | 237 (38.8) |  |
| It is better to use latrine | Agree | 206 (53.9) | 143 (62.4) | 349 (57.7) | 0.37 (0.233, 0.599) |
|  | Disagree | 176 (46.1) | 86 (37.6) | 262 (42.3) | 1 |
| Failing to wash hands may expose you to sickness | Agree | 186 (48.7) | 142 (62.0) | 328 (53.7) | 0.53 (0.362, 0.778) |
|  | Disagree | 196 (51.3) | 87 (38.0) | 283 (46.3) |  |
| I swim, play or wash myself in the river | Never | 61 (16.0) | 62 (27.1) | 123 (20.1) |  |
|  | Sometimes | 154 (40.3) | 85 (37.1) | 239 (39.1) | 1.73 (1.029, 2.911) |
|  | Everyday | 61 (16.0) | 62 (27.1) | 123 (20.1) | 2.37 (1.412, 3.973) |
| If a latrine is available, I will use it | Never | 73 (19.1) | 31 (13.5) | 104 (17.0) | 2.17 (1.244, 3.788) |
|  | Sometimes | 108 (28.3) | 45 (19.7) | 153 (25.0) | 1.59 (0.987, 2.556) |
|  | All the time | 201 (52.6) | 153 (66.8) | 354 (57.9) |  |
| Wash clothes or utensils in open water sources | Yes | 265 (69.4) | 98 (42.8) | 363 (59.4) | 4.02 (2.726, 5.929) |
|  | No | 117 (30.6) | 131 (57.2) | 248 (40.6) | 1 |
| Wash hands when they look or feel dirty | Yes | 171 (44.8) | 131 (57.2) | 302 (49.4) | 1 |
|  | No | 211 (55.2) | 98 (42.8) | 309 (50.6) | 1.83 (1.256, 2.664) |
| Wash hands after defecation | Yes | 149 (39.0) | 108 (47.2) | 257 (42.1) | 1 |
|  | No | 233 (61.0) | 121 (52.8) | 354 (57.9) | 1.36 (0.917, 2.007) |
